# Economic evaluation of an adjunctive intraocular and peri-ocular steroid vitreoretinal surgery for open globe trauma: Cost-effectiveness of the ASCOT randomised controlled trial

**DOI:** 10.1371/journal.pone.0311158

**Published:** 2024-12-16

**Authors:** Victory ‘Segun Ezeofor, Bethany F. Anthony, Lucy Bryning, Edward J. Casswell, Suzie Cro, Victoria R. Cornelius, Catey Bunce, Elizabeth Robertson, Joanna Kelly, Caroline Murphy, Philip J. Banerjee, David G. Charteris, Rhiannon Tudor Edwards

**Affiliations:** 1 Centre for Health Economics & Medicines Evaluation, Bangor University, Bangor, Wales; 2 Vitreoretinal Department, Moorfields Eye Hospital, London, United Kingdom; 3 Imperial Clinical Trials Unit, Imperial College London, London, United Kingdom; 4 Royal Marsden Clinical Trials Unit, Royal Marsden Hospital, National Health Service Trust, London, United Kingdom; 5 King’s Clinical Trials Unit, King’s College London, London, United Kingdom; Tsukazaki Hospital, JAPAN

## Abstract

**Background:**

In the United Kingdom, it is estimated that 5,000 patients sustain eye injuries or ocular trauma requiring hospital admission annually, of which 250 patients will be permanently blinded. This study explores the cost-effectiveness of Adjunctive Steroid Combination in Ocular Trauma (ASCOT) given during surgery versus standard treatment in vitreoretinal surgery in patients with open globe trauma.

**Methods:**

This economic evaluation was embedded alongside the ASCOT RCT (ClinicalTrials.gov Identifier: NCT02873026). We conducted a primary cost-effectiveness analysis from a National Health Service perspective using the proportion of patients who achieved a visual acuity of 10 or more letter improvement on the Early Treatment Diabetic Retinopathy Study (ETDRS) scale as the measure of effect, in developing incremental cost-effectiveness ratios (ICERs). Secondary cost-utility analysis using the EuroQol 5 Dimension 5 Level (EQ-5D-5L) to generate a cost per quality-adjusted life-year (QALY), and a cost-effectiveness analysis using vision-specific quality of life (QoL) was conducted. Sensitivity analyses were also applied to investigate parameter uncertainties.

**Results:**

The sample size of the ASCOT intervention arm and standard care arm of this study was 130 and 129, respectively. The intervention cost per patient was estimated at £132. The proportion of participants with an ETDRS of 10 or more letter improvement was 0.47 for the ASCOT group with a mean cost of £5,526 per patient, while the standard care group had an effect of 0.43 with a mean cost of £5,099 per patient. The ICER value of the primary outcome was £12,178 per 10 or more letter improvement on the ETDRS score. The secondary result in terms of cost per QALYs gained had a probability of 44% being cost-effective at a willingness-to-pay threshold of £30,000/QALY gained.

**Conclusions:**

Though there is no formally accepted cost-effectiveness willingness-to-pay threshold for 10-letter or more improvement, the ASCOT intervention for open globe trauma is a low-cost intervention. The ASCOT intervention is not cost-effective when compared to the standard care in this group and setting. The proportion of patients in the ASCOT intervention arm with 10 or more letter improvement produced some positive results but this is outweighed by the costs.

## Introduction

Although the eyes represent only 0.27% of the human body’s surface area, they are among the most common trauma-exposed areas and can present with severe morbidity [1]. Injuries to the eye, or ocular trauma, are a major cause of visual loss and are estimated to affect 55 million patients worldwide each year, resulting in blindness in 1.6 million eyes [2], and approximately half a million people worldwide end up blind due to ocular trauma [3]. In the United Kingdom (UK), it is estimated that 5,000 patients per year sustain eye injuries severe enough to require hospital admission, and of these, 250 will be permanently blinded in the injured eye [4].

Injuries to the eye, or ocular trauma, are most likely to occur in men under 40 years of age and are often caused by work-related accidents, falls, sports, assaults, and traffic incidents [3, 5]. In many instances, ocular trauma can be treated in primary care, and treatments may include cycloplegic medication, antibiotic treatment, and eye shields/patches; however, more severe forms of ocular trauma will often require immediate investigation in secondary care and surgical treatment [6]. Eye injuries cost over $300 million per year in the United States (US) [7], which equates to an annual cost to the UK economy (for which no comparable data exist) of £37.5 million (direct and indirect costs) [8, 9].

Open globe injuries are a type of ocular injury that includes penetrating and or perforating injuries (e.g. a full-thickness wound of the eyewall with a sharp or pointed object), intraocular foreign bodies, as well as globe ruptures [10]. Preferred management practices for open globe injuries vary widely; to ensure the highest standard of care for all patients, evidence-based international guidelines for the treatment of these injuries are needed [11].

Several studies investigating proliferative vitreoretinopathy (PVR), the most common cause of retinal detachment associated with a worse visual outcome [12], suggested that corticosteroids can downregulate the pathobiological process of PVR development [13–15]. Several studies indicated that the adjuvant use of corticosteroid was effective in open globe trauma [16]. There has been inconsistent evidence on the effect of dexamethasone in vitreoretinal surgery, a study by Banerjee et al., 2017 suggests a positive effect [17] while Koerner et al., 2012 does not show a positive effect [18]. This trial aims to test the hypothesis that adjunctive triamcinolone acetonide (TA), given at surgery can improve outcome of open globe trauma [19].

The clinical effectiveness of these interventions is very important but so is their health economics evaluation. There have been several cost-effectiveness studies on eye conditions such as cataract surgery [20] and other interventions relating to eye health [20–22], but none explored the cost-effectiveness of medical interventions to treat ocular trauma or injury. Concerning the UK, we did not find any National Institute for Health and Care Excellence (NICE) guidance and economic evaluations on this topic. A study like this will benefit from an economic evaluation, as this will investigate if its cost-to-benefit ratio will be cost-saving to the NHS or cost-effective to the stakeholders or patients. A reduction in service use, faster recovery rate, and better visual quality of life in comparison to standard care will be better value for money.

There seems to be a gap in the literature regarding the cost-effectiveness of ocular trauma interventions. We aim to fill this gap by conducting an economic evaluation of the cost-effectiveness of adjunctive steroid administered at the time of surgery compared with standard treatment (no adjunctive treatment) in vitreoretinal surgery for patients with open globe trauma. This economic evaluation was conducted alongside a phase 3, multi-centre, double-masked randomised controlled trial (RCT) of adjunctive intraocular and peri-ocular steroid (triamcinolone acetonide) in patients with open globe ocular trauma [23]. Though data from the RCT suggests no clinical benefits [19] in this study, we examine the cost and cost-effectiveness of the intervention compared to standard treatment.

## Methods

### Trial and data collection

The Adjunctive Steroid Combination in Ocular Trauma (ASCOT) randomised controlled trial had a 1:1 randomisation of 300 adult patients with open-globe trauma from 28 vitreoretinal surgery centres throughout the UK. The ASCOT intervention group was to receive adjunctive intraocular and peri-ocular steroid (triamcinolone acetonide) included in the standard surgery. In contrast, the standard care (control group) had surgery without adjunctive treatment, just standard surgery alone.

The medication administered to the participants in the ASCOT treatment/intervention arm was as follows:

1. 4mg/0.1ml triamcinolone acetonide: This was administered into the vitreous cavity by the operating surgeon at the end of the procedure.
2. 40mg/1ml of triamcinolone acetonide: This was administered into the subtenons space at the end of the procedure.

For more details on the recruitment, eligibility criteria, and intervention treatment see Casswell et al., [19].

The standard care and the intervention groups received standard surgical treatment and routine preoperative and postoperative treatment and care.

The primary outcome was the proportion of participants with an improvement in corrected visual acuity in the study eye, defined as having a change of 10 or more letters in the Early Treatment Diabetic Retinopathy Study (ETDRS) score. Data for the economic evaluation were collected alongside other measures at baseline and follow-up (at 3 months and 6 months) [24]. Data on resource use was also collected along with medications, dosage, and frequency.

Ethical approval was granted by NRES Committee London Central (14/LO/1428) on 14 September 2014. The trial was open to recruitment from 1^st^ December 2014 until 31^st^ March 2020. Each participant gave written informed consent to participate in the study before taking part. Further trial details are provided elsewhere [19, 25].

### Economic evaluation

The economic evaluation time horizon was 6 months. The cost-effectiveness model of the intervention trial was not conducted beyond the trial period, and since the period was less than 12 months, no discounting was applied to either cost or health outcomes. The primary aim of the economic analysis was to establish the cost per 10 or more letter improvement in ETDRS score gained at 6 months from a National Health Service (NHS) perspective in compliance with the study protocol [26]. We also conducted secondary analyses investigating the cost per quality-adjusted life-year (QALY) gained at 6 months following the National Institute for Health and Care Excellence guideline [27, 28]. Further secondary analyses were investigated for the proportion of patients who had achieved 10 or letter more improvement in ETDRS using the cost per QALY and visual quality of life (VQoL) using the VFQ-25 scale.

#### Costs

In this study, all resource use costs were examined from an NHS perspective in terms of the identification, measurement, and valuation of costs [29, 30]. Resource use and cost information were collected on secondary care and prescribing from hospital-held patient records by researchers at each ASCOT trial centre. The Client Service Receipt Inventory (CSRI) as part of the Case Report Form (CRF) was informed by the DIRUM database [31, 32].

The cost associated with this trial can be classified into two: the intervention cost and the cost incurred during the trial period (resource use cost).

The resource use costs were grouped into three categories: hospital-based service cost, community-based service cost, and medication cost. These costs were collected for the preoperative care at baseline and each of the follow-up time points. Costs incurred directly or associated with the usual care during the trial period such as GP visits, therapy sessions, emergency visits, and check-ups were captured using the CSRI. Drug costs were obtained from the Prescription Cost Analysis [33].

The intervention cost consisted of additional costs incurred for the ASCOT surgical procedure that would not be in the standard care package. Items included in the intervention costs were the cost of triamcinolone acetonide, additional time required to administer the intervention and staff costs. These are add-on costs to the standard package.

The valuation of the costs was derived from the Personal Social Services Research Unit [34], and the NHS reference costs [33, 35], and where these costs were not referenced, experts’ opinions were applied. All costs were reported in Pounds Sterling (£) and inflated to costs year 2022/23 where necessary, using the PSSRU guide for inflation of hospital resources [34].

#### Outcomes

The primary outcome measure for the economic analysis of the trial was the cost per the proportion of patients who achieved an ETDRS of 10 or more-letter improvement (also known as the incremental cost-effectiveness ratio (ICER)) in corrected visual acuity at six months from an NHS perspective. A 10-letter improvement in visual acuity (measured using validated ETDRS vision charts at a starting distance of 4 metres) represents the widely accepted minimum clinically meaningful difference in research studies of eye disease [36–45]. We also conducted secondary cost-utility analyses using the patients’ QALY values. This was further investigated considering only patients with at least a 10-letter or more improvement in visual acuity. The VFQ-25 questionnaire was also used to evaluate the vision-related quality of life (VQoL) of the patients with an ETDRS score of 10 or more letter improvement.

QALYs gained were used as a measure of health utility calculated by ‘weighting’ each period of follow-up time by the value corresponding to the health-related quality of life (HRQoL). The QALY value was assessed using a crosswalk mapping of the EQ-5D-5L questionnaire [46] to the EQ-5D-3L. The EQ-5D is a generic, preference-based HRQoL measure [47, 48] while the ETDRS and VFQ-25 are disease-specific outcome measures of VQoL [49, 50].

The changes in outcome measures were calculated using the area under the curve method, with a linear change between assessment points [51].

#### Data

As the intervention was a one-off treatment at the time of randomisation and the two follow-up appointments followed usual clinical care, we anticipated a low percentage of missing primary outcome data. It was therefore anticipated that missing data would be missing at random (MAR) [52]. Multiple imputation (MI) using the hot-deck method estimation was used for handling missing outcome data [53, 54], taking into account covariates including utilities, age, and gender. Each imputed data was analysed, and the estimates were pooled to generate a mean value. No donor limits were applied due to the low percentage of missing data and sufficient sample size. The evaluated cost-effectiveness of the ASCOT intervention in comparison to the standard/usual care was conducted using Microsoft Excel for Office 365.

#### Sensitivity analyses

We undertook both deterministic and probabilistic sensitivity analysis to test the uncertainty of the findings. These analyses were used to test how sensitive the results were to basic assumptions, methods of analysis, and parameters [55–57]. Varying the assumptions, the stability of the findings was tested, and the confidence of the results was accounted for. One-way sensitivity analysis was represented using the tornado plot. The tornado plot shows changes in the value of the ICER when a variable influencing the outcome of the health economic evaluation is varied.

The probabilistic sensitivity analysis (PSA) was undertaken using the Monte Carlo simulation with 5,000 samples to determine the level of sampling uncertainty around the ICER value using appropriate distributions. These results were represented in a cost-effectiveness plane and cost-effectiveness acceptability curves (CEAC) were plotted.

#### Health economics analyses

We analysed the cost-effectiveness of the ASCOT intervention in eyes undergoing vitreoretinal surgery for ocular trauma compared to surgery alone in terms of changes in visual acuity. For this analysis, the following was needed:

(a) Recording the intervention add-on cost for the surgery.

(b) Recorded study participant primary and secondary care health service use and social care use over the 6 month follow-up period (using a research nurse interviewer administered CSRI, costed using National unit costs), and making use of routine hospital data on surgical and post-operative care as part of the CRF.

(c) Conducted a primary cost-effectiveness analysis (using the trial primary outcome measure of the proportion of patients with visual acuity ≥10 letter improvement in ETDRS score, as our measure of effectiveness).

(d) A secondary cost-utility analysis to explore the cost per QALY of the ASCOT intervention as compared with usual treatment relative to the £20,000 -£30,000 per QALY NICE payer threshold [58–60]. NICE currently uses this threshold to determine whether the health benefits provided by a new drug or healthcare intervention is greater than the health likely to be lost as services are displaced to accommodate for the new intervention [58, 61].

(e) The sensitivity analysis generated CEACs to communicate to policymakers the probable cost-effective ratio at various willingness-to-pay (WTP) thresholds.

The incremental net monetary benefit (INMB) of adopting ASCOT treatment instead of the standard care treatment was also produced to describe the extent to which the intervention is deduced to be cost-effective [62, 63].

For the economic analysis, patients who withdrew from the trial were not included in the health economic analysis. The reporting of our economic analysis draws on the reporting guidance from the CHEERS checklist shown in S1 Appendix [64, 65] and NICE reference case guidance [28, 66].

## Results

### Analysis of baseline data

From the 280 patients recruited into the trial (143 patients were randomised to the ASCOT intervention group; 137 patients were randomised to the standard care group). At the 6 month follow-up, there was over 92% completion rate (259 patients: 130 patients in the ASCOT treatment arm; 129 patients in the standard care arm). The demographic characteristics were similar across both groups (Table 1).

**Table 1:** Demographic and baseline characteristics of patients in the ASCOT intervention and standard care group.

	Standard Care	ASCOT
	N = 137	N = 143
	n (%)	n (%)
Gender		
Male	123 (90%)	123 (86%)
Female	14 (10%)	20 (14%)
Ethnicity		
White	113 (82%)	120 (84%)
Black	11 (8%)	9 (6%)
Asian	7 (5%)	11 (8%)
Mixed	3 (2%)	0 (0%)
Other	3 (2%)	3 (2%)
Injured Eye		
Right	67 (49%)	70 (49%)
Left	66 (48%)	72 (50%)
Both	4 (3%)	1 (1%)
ETDRS completion (%)		
Baseline	96	95
3 months	95	94
6 months	98	99
EQ-5D completion (%)		
Baseline	89	92
3 months	81	81
6 months	83	87
VFQ-25 completion (%)		
Baseline	90	93
3 months	80	82
6 months	83	87

The outcome data for each treatment arm had a high completion rate. The ETDRS at baseline had a 96% completion rate for the control arm while the ASCOT intervention arm had a 95% completion rate. This was similar at the 3 month follow-up where there was a 1% difference in the completion rate. At 6 months there was a 99% completion rate for the ASCOT arm of the study while the usual care had a 98% completion rate. For other measures, there was a high completion rate, with the lowest being 80% at the 3 month assessment point for the VFQ-25 (Table 1).

### Costs

The breakdown and components of the ASCOT intervention cost are described in Table 2. The intervention cost is directly linked to the additional cost of implementing the ASCOT intervention. The time allocated to preparing and implementing the additional service was estimated to be 30 minutes. This procedure was led by a surgical consultant and assisted by two Nurses. All staff costs are shown in Table 2. The total cost for the ASCOT intervention was estimated at £132 per patient.

**Table 2:** Cost of Intervention. Additional costs incurred for the ASCOT intervention.

	Items	Cost		Source/ Information
		**4mg/0.1ml**	**Total**	
1	Time to prepare and administer	30min		Obtained from consultations with experts and consultants
2	Cost Consultant	£143	£71.5	Hospital-based doctors. Unit cost per hour (consultant surgical) page 101. Includes qualification costs [34].
	Number of Nurses	2		Obtained from consultations with experts and consultants
3	Nurse	£59	£59	Hospital-based nurse. Unit cost per hour (Band 6) page 97. Includes qualification costs [34].
4	Cost of Medication			
	**4mg/0.1ml**	£0.149	£0.149	[67]
	**40mg/1ml**	£1.49	£1.49	[67]
5	Medical Equipment			Obtained from consultation with experts and consultants
	Cost of Syringe	£14.81 for 100 pieces	£0.148	https://www.primarycaresupplies.co.uk/syringe-1ml-1-x-box-of-100/ [assessed 05/02/24]
	Gloves		--	Provided by the Hospital and applicable for both arms of the intervention. Not an additional cost due to intervention.
	Ophthalmic scissors		--	
	Universal Wire Speculum		--	
	Plain Forceps		--	
	Wescott’s Spring Scissors		--	
	Steven’s Cannula		--	
	27 Gauge Visco Cannula		--	
	Headband light		--	
	**Grand Total Cost**		**£132.29**	

The cost incurred for the standard surgical procedure across the NHS for both groups was the same. To avoid double counting, the health economics analysis assumes a zero cost for the standard surgical package. The standard care arm had no additional cost.

Table 3 contains the breakdown of the resource use cost at each assessment time point. This is divided into hospital-based service cost, community-based service cost, and medication cost. Though the hospital-based service costs were similar for both arms at all time points (baseline and follow-up), the ASCOT intervention arm had a slightly higher cost at all assessment time points except at the 3 month follow-up point. The community-based service cost was also similar across both arms of the study with the ASCOT intervention arm having slightly higher costs. For the medication costs the standard care arm had a total higher cost than the ASCOT intervention arm.

**Table 3:** Summary of the mean (SD) costs by patients (N = 259) in the ASCOT trial over 3 time points (baseline, 3 months, and 6 months).

Cost components	Standard Care (£) (n = 129)	ASCOT Intervention (£) (n = 130)	Difference (£)
**Baseline** ^**+**^			
Hospital-Based Service cost	245.61	319.80	74.19
Community-Based Service cost	943.16	1,087.25	144.09
Medication Cost	1,581.59	1,694.92	113.32
Total	2,770.36	3,101.96	331.60
**3 months**			
Hospital-Based Service cost	276.56	272.58	-3.98
Community-Based Service cost	308.45	412.28	103.83
Medication Cost	721.63	540.45	-181.18
Total	1,306.64	1,225.30	-81.34
**6 months**			
Hospital-Based Service cost	244.65	329.01	84.36
Community-Based Service cost	183.02	254.10	71.08
Medication Cost	594.07	483.95	-110.12
Total	1,021.75	1,067.07	45.32
**GRAND TOTAL**	**£5,098.74**	**£5,394.33**	

^+^Baseline costs were collated from preoperative costs [19, 26].

The preoperative resource use cost (baseline cost) had the highest cost of £3,102 and £2,770 for the ASCOT Intervention and standard care arm respectively. The resource use costs had a continuous decline of over 50% postoperation, at the 6 month follow-up, the resource use costs was £1,067 and £1,022 for the ASCOT Intervention and standard care arm respectively. Over the entire trial, the standard care group had a mean cost of £5,099 while the ASCOT intervention arm had a mean cost of £5,394. This brings the total economic cost (intervention cost plus resource use cost) of the ASCOT intervention to £5,526. The unit costs and descriptives on the resource use are in Table 3 and S1–S3 Tables.

### Outcome measures

The proportion of participants with an improvement of 10 or more letters ETDRS score in the ASCOT intervention arm was 47%, while the standard care arm had a proportion of 43%, see Table 4. The mean EQ-5D score at baseline was 0.69 for both arms of the study. At the 6 month follow-up, the mean EQ-5D score was 0.77 and 0.78 for the ASCOT and the standard care arm respectively (Table 4). The scores for the VQoL using the VFQ-25 were 66.95 and 64.62 at baseline and 74.19 and 73.49 at the 6 month final follow-up for the standard care and ASCOT intervention arm respectively. At each assessment point, the EQ-5D and the VFQ-25 scores were similar between groups. A more detailed breakdown of the outcome measures is described in Casswell et al, 2023 [19].

**Table 4:** Outcome measures and proportions at baseline and follow-up assessment time points.

	Standard care		ASCOT Intervention	
**Proportions**				
**ETDRS**	**(n = 129)**		**(n = 130)**	
≥ 10	56	(43%)	61	(47%)
< 10	73	(57%)	69	(53%)
	**Mean**	**SD**	**Mean**	**SD**
**EQ-5D**				
Baseline	0.69	0.26	0.69	0.28
3 months	0.78	0.23	0.77	0.20
6 months	0.78	0.24	0.77	0.24
**VFQ-25**				
Baseline	66.95	21.45	64.62	23.06
3 months	71.94	19.34	69.77	18.33
6 months	74.19	22.29	73.49	21.61

### Cost-effectiveness analysis

The result of the cost-effectiveness analysis in comparing the ASCOT treatment in the eyes undergoing vitreoretinal surgery for open globe trauma versus the standard procedure is captured in Table 5. The primary analysis resulted in an incremental cost of £428 between the ASCOT treatment and the standard care, the ASCOT intervention group had a higher cost of £5,527. The patients with 10 letters or more improvement in each arm had higher costs, of which the ASCOT intervention arm had the largest subgroup mean cost of £6,446 while the standard care patients had a subgroup mean cost of £5,268. The mean cost for the patients within each group with less than 10 letter improvement was the ASCOT arm with a subgroup mean cost of £4,713, and the standard care had a subgroup mean cost of £4,968, see S4 Table for more details.

**Table 5:** Cost-effectiveness results for the ASCOT intervention compared to standard care.

				ICER	Probability of cost-effectiveness			INMB	
	ASCOT	SC^d^	Incremental	Cost/Effect	£20,000	£30,000	P^e^	£20,000(95% CI)	£30,000(95% CI)
**Primary Outcome**									
ETDRS									
ETDRS^a^ ≥ 10	0.47	0.43	0.04	£12,178/ETDRS (≥10)	0.58	0.63	£11,700	269(-2523,3063)	621 (-3322,4528)
Mean Cost	£5,527	£5,099	£428						
**Secondary Outcome**									
QALY-EQ-5D									
QALY	0.04	0.03	0.01	£42,289/QALY	0.38	0.44	£42,800	-239(-1687,1269)	-140(-1690,1435)
Mean Cost	£5,527	£5,099	£428						
QALY^b^ (ETDRS≥ 10)									
QALY	0.05	0.03	0.01	£32,900/QALY	0.41	0.48	£33,900	-176(-1700,1378)	-46 (-1724,1637)
Mean Cost	£5,527	£5,099	£428						
VQoL-25^c^ (ETDRS≥ 10)									
VQoL	0.34	0.35	-0.01	Dominated	0.23	0.22	- -	-606 (-2152,974)	-690(-2391,1030)
Mean Cost	£5,527	£5,099	£428						

^a^This represents the proportion of patients with an ETDRS improvement of 10 letters or more.

^b^This represents the QALY value derived from the EQ-5D-5L score cross-walked to the EQ-5D-3L of patients who had an improvement ETDRS score ≥ 10 letters or more.

^c^This represents the VFQ-25 score of patients who had an improvement ETDRS score ≥ 10 letters or more improvement.

^d^SC = Standard Care.

^e^This is the willingness-to-pay threshold at a 50% probability of cost-effectiveness.

The proportion of patients satisfying the primary outcome (ETDRS ≥ 10 letter improvement) is shown in Tables 4 and 5. The ASCOT arm had 47% of the patients achieving a 10-letter or more improvement while only 43% of the standard care arm achieved this. This resulted in a mean ICER value of £12,178 per 10 or more letter improvement as shown in Table 5.

### Sensitivity analysis

**Fig 1:**
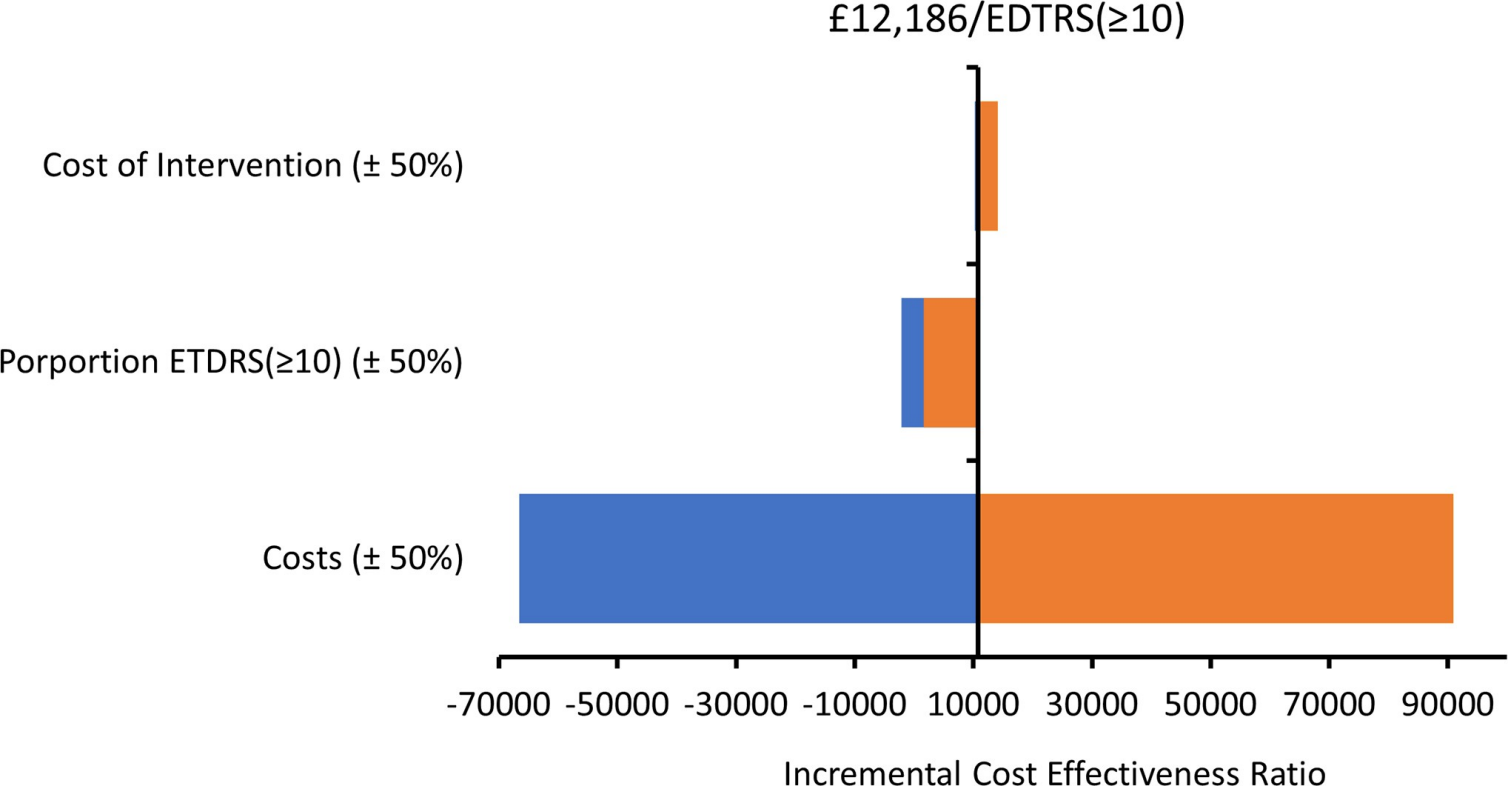
Tornado diagram for a multiple 1-way sensitivity analysis. Each variable is analysed for a ±50% variability.

A deterministic sensitivity analysis results are shown in the tornado plot in Fig 1. In the tornado plot, we varied three parameters (cost of intervention, probability of achieving an EDTRS ≥10 letter improvement, and the mean total cost of the intervention arm) by ±50% of its original value to investigate the change in ICER value. The greatest influencer of the ICER value was the mean total cost. A reduction in the intervention cost by 50% gives a lower ICER of -£66,493 per EDTRS ≥10 letter improvement while an increase of 50% results in an ICER value of £90,866 per ETDRS ≥10 letter improvement.

**Fig 2:**
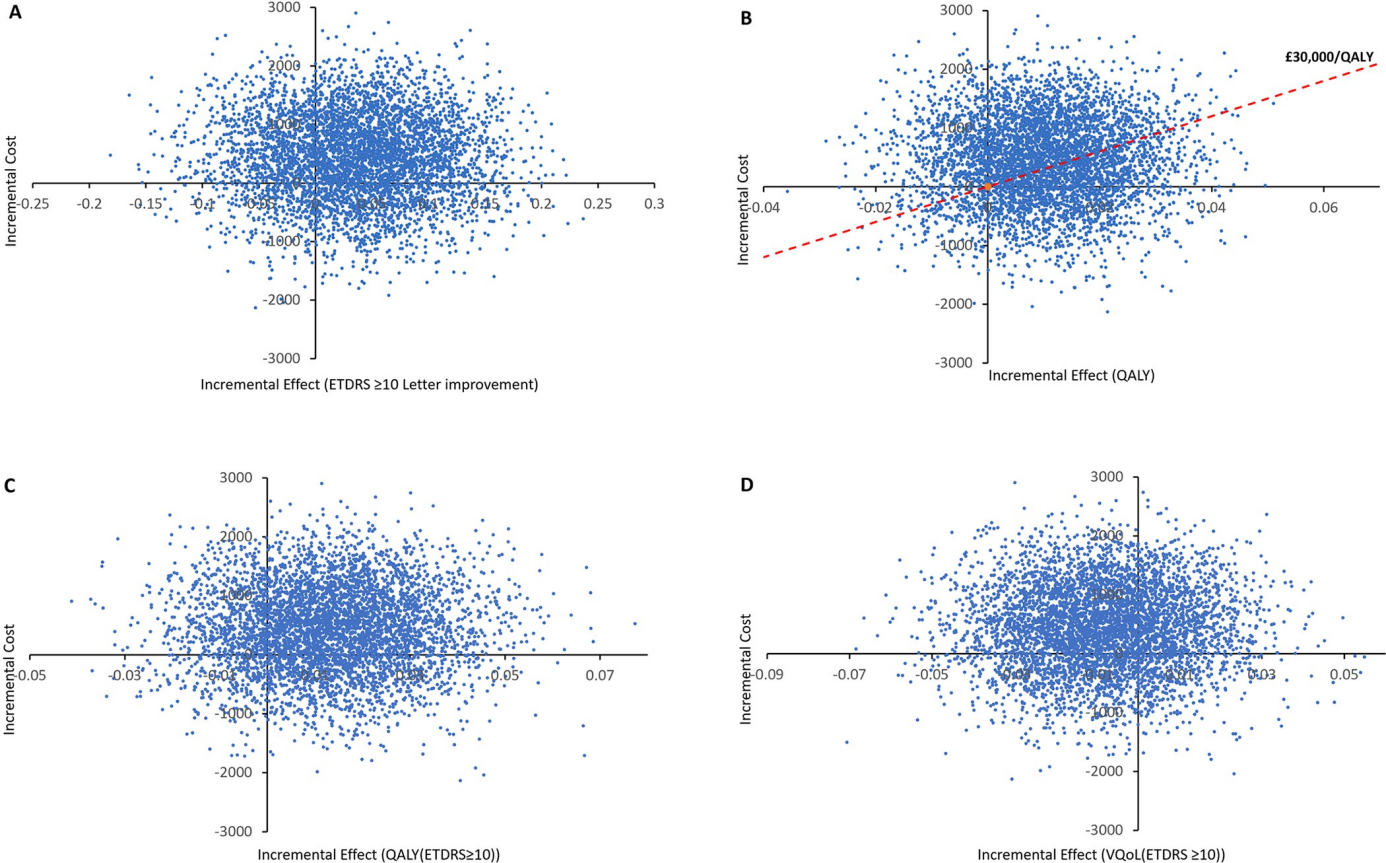
A scatter plot of 5000 simulations displaying the incremental cost-effectiveness ratio of the ASCOT intervention compared to standard care. (A) ICER of at least an ETDRS 10-letter improvement, (B) ICER of the cost per QALYs gained, (C) ICER of the cost per QALYs per 10 or more letter gained, and (D) ICER of the VQoL.

A simulation of 5,000 ICER samples is shown in Fig 2A on the cost-effectiveness plane where the ASCOT intervention is compared to the standard care in the cost-effectiveness analysis, (see S4 Table for distribution applied to parameters). The ICER simulations are distributed over the four quadrants of the scatter plot but the north-east quadrant was more populated which shows that the ASCOT intervention was more costly and more effective than the standard care. This matches the result obtained from Table 5.

**Fig 3:**
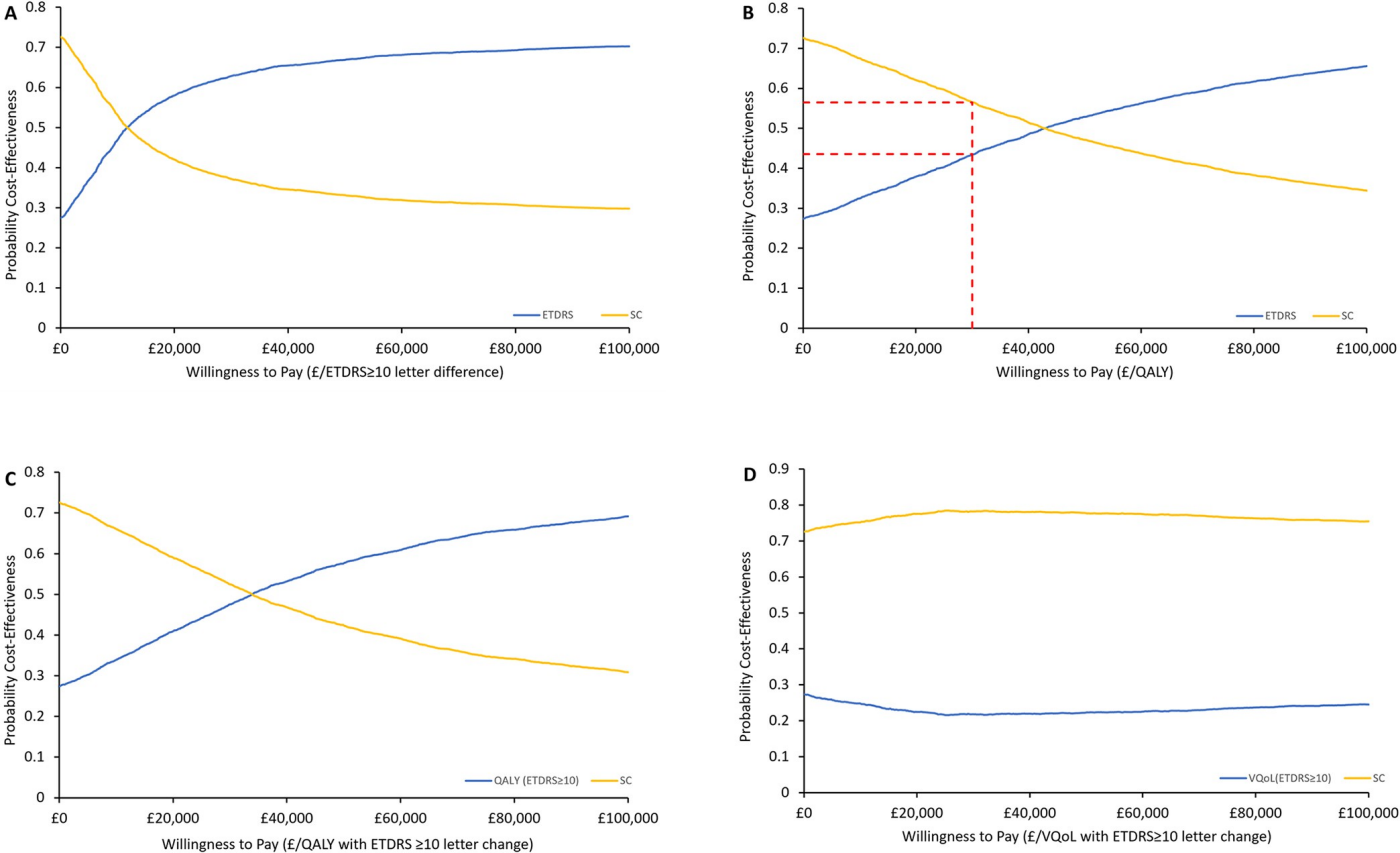
Cost-effectiveness acceptability curve of the ASCOT trial. (A) Primary outcome measure, ETDRS of 10 or more letter improvement (B) cost per QALYs gained, (C) cost per QALY per ETDRS of 10 letter or more improvement, and (D) cost per VQoL per ETDRS of 10 letter or more improvement. SC = Standard care, VQoL = Visual quality of Life.

Investigating further with the simulated samples, the CEAC curve is generated (Fig 3A), showing the probability of the ASCOT intervention being cost-effective at different willingness-to-pay thresholds. The plot shows that the ASCOT intervention is cost-effective at a willingness-to-pay threshold of about £12,000 per ETDRS of 10-letter or more improvement.

**Fig 4:**
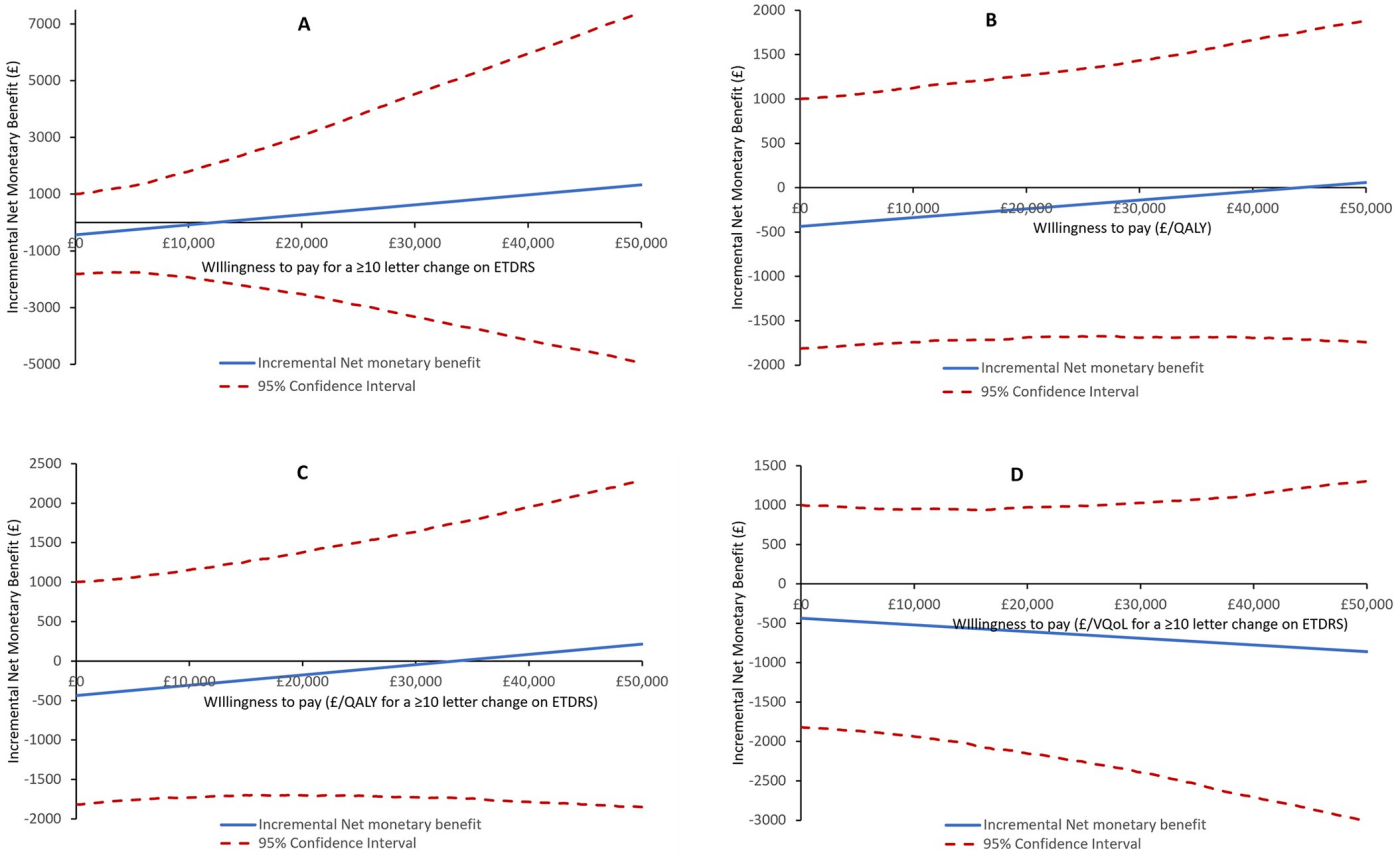
Incremental net monetary benefit plot. (A) Primary outcome measure, ETDRS of 10 or more letter improvement (B) cost per QALYs gained, (C) cost per QALY per ETDRS of 10 letter or more improvement, and (D) cost per VQoL per ETDRS of 10 letter or more improvement. SC = Standard care, VQoL = Visual quality of Life.

The associated mean INMB at various willingness-to-pay thresholds was also investigated as part of the probabilistic sensitivity analysis. The INMB curve (Fig 4A) shows the net benefit changes from negative to positive after a willingness-to-pay threshold of £12,300 per EDTRS ≥ 10 letter improvement. This suggests that the ASCOT intervention group would result in an average net economic benefit after this threshold value. However the confidence intervals of the INMB curve do not cross zero, illustrating that the 95% confidence limit of the ICER could not be defined, and therefore, neither the ASCOT intervention nor the standard care can be deduced to be the more cost-effective strategy with a high level of confidence (see Fig 4A).

### Secondary results

The secondary analysis using the cost and outcome results for the QALY value was investigated and presented in Tables 4 and 5. We analysed the costs, benefits, and INMB for both arms of the study. The QALY value in the ASCOT intervention arm was slightly higher with a value of 0.01 compared to the standard care arm. This gave an ICER value of £42,289/QALY.

We also analysed the QALY and VQoL of patients who achieved a minimum ETDRS score improvement of 10 letters or more (Table 5). In this analysis, the QALY value for the ASCOT intervention was 0.05 while the standard care arm had a QALY value of 0.03 leading to an ICER value of £32,900 per QALY per ETDRS of 10 or more letter improvement. For the VQoL derived from the VFQ-25, for patients with a 10 or more letter improvement ETDRS score, the standard care had a higher VQoL mean effect value of 0.35, and the ASCOT intervention arm had a VQoL value of 0.34, this led to the ASCOT intervention being dominated by the standard care intervention (see Table 5).

A probabilistic sensitivity analysis of the secondary measures was also undertaken to generate 5000 simulated cost-effectiveness samples as shown in Fig 2B–2D. In all simulations, the ICER samples were distributed over the four quadrants. In Fig 3B and 3C, the simulations for the QALY values had slightly higher density in the north-east quadrant. This showed that the ASCOT intervention was more effective and expensive than the standard care. In Fig 2D, the scatter plot for the VQoL of patients with at least an ETDRS of 10-letter improvement shows a more densely populated north-western region. This implies that the ASCOT intervention was more costly but less effective, showing standard care as the preferred option.

Fig 3B–3D show plots of the probability of the cost-effectiveness of the secondary measures at different willingness-to-pay thresholds. The CEAC curve in Fig 3B shows a probability lower than 0.5 for the ASCOT intervention below a willingness-to-pay threshold of £42,800 per QALY. The result was similar when comparing the probability of the willingness-to-pay threshold per QALY for patients who had a minimum ETDRS score of 10 letter or more improvement (£33,900 per QALY per ETDRS ≥10 letter improvement), see Fig 3C. The CEAC curve of the VQoL in Fig 3D shows that the ASCOT intervention had less probability of being cost-effective than standard care. The ASCOT intervention had a probability below 30% of being cost-effective at all willingness-to-pay thresholds, that is from £0/VQoL per ETDRS of 10 or more letter improvement to £100,000/VQoL per ETDRS of 10 or more letter improvement. This shows that standard care has a higher probability of being cost-effective in comparison to the ASCOT intervention when considering the VQoL.

The CEAC curve confirms the results previously obtained from the scatter plot of the cost-effectiveness plane, that there is little or no advantage of implementing the ASCOT intervention over and above standard care when considering the QALY values gained. In terms of VQoL, irrespective of the willingness-to-pay thresholds, the probability of the standard care being cost-effective was better than the ASCOT intervention.

For the QALY outcome, the INMB at a willingness-to-pay of £0 was -£436 (95% CI -£1,816, £1,002), and at a willingness-to-pay of £20,000 was -£239 (95% CI -£1,687, £1,269). The corresponding INMB of the ASCOT intervention at £30,000 also was negative, -£140 (95% CI -£1,690, £1,435), meaning that the ASCOT intervention does not provide value for money compared to the standard care at the £30,000 per QALY willingness-to-pay threshold (Fig 4 and Table 5). Similarly, for the VQoL, the INMB was also negative, -£606 (95% CI -£2,152, £974) and -£690 (95% CI -£2391, £1030) at the £20,000 and £30,000 willingness-to-pay threshold respectively (see Fig 4 and Table 5). This suggests that the ASCOT intervention was accumulating more costs for lower benefits than the standard care.

For the secondary outcome measures, the costs outweighed the benefits of the ASCOT intervention resulting in an average net economic loss. The ICER, CEAC, and INMB results all had the same conclusion.

## Discussion

The ASCOT intervention was a low-cost intervention for open globe trauma, estimated at £132 per patient. This accounted for the higher difference in cost between the ASCOT intervention and standard care arms of the trial. In our primary analysis, the proportion of participants who achieved an ETDRS ≥10 letter improvement was 0.47 in the ASCOT intervention group with a mean cost of £5,527, while the standard care group had a mean cost of £5,099 and a proportion of 0.43 participants with an ETDRS ≥10 letter improvement, see Table 5. The primary outcome measure (ETDRS) had an ICER of £12,178 per ETDRS score of 10 letters or more improvement. The sensitivity analysis conducted strengthens the position that the value obtained from the base case analysis is consistent as the ICER plot for the primary outcome shows denser simulations in the north-eastern quadrant of the scatter plot, (see Fig 3A). The CEAC curve in Fig 4A gives a similar result to that in Table 5, a probability of having a 50% probability of the ASCOT intervention being cost-effective has a willingness-to-pay threshold value of approximately £11,700 per ETDRS score ≥10 letter improvement. The result of the deterministic analysis, the tornado plot shows three high-impacting parameters of the cost-effectiveness result. The parameter with the highest impact is the resource use costs in which a 50% reduction will amount to a cost-savings for the NHS, see Fig 1.

In our secondary analysis, The ASCOT intervention showed little or no improvement in the utility measures. The ASCOT intervention arm had a QALY value of 0.04 QALYs while the standard care arm had a QALY value of 0.03 giving a difference of 0.01 between the two study arms. A similar difference of 0.01 was also obtained for the QALY value of patients who had an ETDRS score of 10 or more letter improvement. For the visual function, the VQoL measure was 0.01 less in the ASCOT care arm. None secondary outcome measures had a cost-effectiveness ratio below the £30,000 per QALY willingness-to-pay threshold. This signified that the ASCOT intervention was not cost-saving or cost-effective following the NICE guideline. For the VQoL, the ASCOT intervention was dominated by the standard care, showing that the standard care was less expensive and provided more benefits.

The outcome measures ETDRS and VFQ-25 are not preference-based measures, but the EQ-5D is a preference-based measure that can be compared with the NICE £20,000 to £30,000 per QALY threshold, this gives this study a multi-dimensional assessment to its economic evaluation. In Fig 3, the ICER scatter plots show the simulated points scattered over the four quadrants, indicating that there is no 100% certainty that either the ASCOT intervention or the standard care was always more cost-effective. The distribution of the simulated ICER points was slightly more in the north-east quadrant of the scatter plot (except for the VQoL) which shows that the ASCOT intervention had the possibility of being cost-effective, but this was at a higher WTP threshold value above the £30,000 per QALY recommended by NICE. This result is further emphasised in the CEAC curves in Fig 4B.

The CEAC plot (Fig 4B) shows that the intervention has a low probability of being cost-effective with approximately 44% at a willingness-to-pay threshold of £30,000 per QALY gain compared to the standard care. The result for the EQ-5D not being significantly different agrees with the study from Gazzard et al., [68]. The CEAC plot for the VQoL showed that ASCOT intervention was dominated by the standard care, and the probability of being cost-effective for all willingness-to-pay thresholds was below 30%.

### Strengths and weaknesses

A strength of this economic evaluation is that most parameters are based on the evidence available from this ASCOT trial, as studies have shown that models developed by incorporating data from various sources are often not adequate [69, 70]. By conducting the sensitivity tests, we further emphasize the robustness of the economic evaluation.

This is the first study that evaluates the cost-effectiveness impact of adjunctive intraocular and peri-ocular steroid (triamcinolone acetonide) to standard treatment. This forms a foundation for future studies in this area.

The broad inclusion criteria and the lack of specification for vitrectomy was observed as a limitation in this trial [19]. Future PVR trial design needs clarity on classification or degree of trauma categorisation.

To further strengthen the transparency in this economic evaluation, the CHEERS checklist [64, 65] was strictly followed and complied with.

### Comparison with other studies

Studies such as [71] who explored adjunctive steroid therapy versus antibiotics alone for acute endophthalmitis after intraocular procedure stated that they did not conduct formal cost‐effectiveness analyses, but compared the costs associated with the interventions when data were available. In other adjunctive steroid therapy, only clinical effectiveness was investigated [72–74] hence this ASCOT trial provides key evidence for the clinical and cost-effectiveness [19] of adjunctive steroid therapy versus standard treatment.

### Future research

This is a low-cost intervention however, it did not produce a significant benefit to impact the health economic outcome, and hence the ASCOT intervention was not cost-effective in terms of QALY gains.

The evidence from the evaluation showed that there were no cost-savings or cost-effectiveness benefits from ASCOT intervention in open globe trauma cases. Other therapeutic agents may hold potential but future clinical trials will need to be designed to allow the optimal conditions to test specific treatments [75]. Differing agents with alternate modes of delivery may be more appropriate for other trauma subtypes such as globe ruptures which have a high rate of Proliferative Vitreoretinopathy (PVR) [12]. Timing may be of central importance here, early intervention with surgery and or therapeutic adjuncts is likely to give the best chance of success and of testing the adjunct in trials. Trial designs will therefore need to have a clear focus on disease categories and surgical protocols will need to be tightly drawn up both to surgical technique and the timing of intervention.

## Conclusions

Adjunctive intraocular and peri-ocular steroid vitreoretinal surgery for open globe trauma is a low-cost intervention; however, it did not produce significant outcomes of effects to outweigh the additional cost. The proportion of patients in the ASCOT intervention arm with at least a 10-letter improvement using the ETDRS score produced some positive. Evidence from this study indicates that implementing the ASCOT Intervention in the eyes undergoing vitreoretinal surgery for open globe trauma was not cost-effective using the NICE threshold guideline of £30,000 per QALY gained. In the treatment of eye injury, adding triamcinolone acetonide, to treatment did not seem to be a good value for money.

## Supporting information

S1 Table Summary of hospital-based service use mean (SD) costs by patients (N = 259) in the ASCOT trial over 3 time-points (baseline, 3 months and 6 months). (DOCX)

S2 Table Summary of community-based service use mean (SD) costs by patients (n = 259) in the ASCOT trial over 3 time-points (baseline, 3 months and 6 months). (DOCX)

S3 Table Types and cost of medication and drugs administered to patients during the trial. (DOCX)

S4 Table Distribution for the probabilistic sensitivity analysis. (DOCX)

S1 Appendix CHEERS checklist. (DOCX)

S1 Dataset (XLSX)
