# Supplementary material for: Economic evaluation of an adjunctive intraocular and peri-ocular steroid vitreoretinal surgery for open globe trauma: Cost-effectiveness of the ASCOT randomised controlled trial
Source: PLoS One. 2024 Dec 16;19(12):e0311158. doi: 10.1371/journal.pone.0311158 (PMC11649106; doi:10.1371/journal.pone.0311158)
Supplement: S1 Table — (DOCX) [file pone.0311158.s001.docx]

# Supporting information

**S1 Table. Summary of hospital-based service use mean (SD) costs by patients (N=259) in the ASCOT trial over 3 time-points (baseline, 3 months and 6 months).**

|  | **Standard Care (£)**  **(n = 129)** | | | **ASCOT Intervention (£)**  **(n = 130)** | | |
| --- | --- | --- | --- | --- | --- | --- |
|  | **Baseline** | **3 months** | **6 months** | **Baseline** | **3 months** | **6 months** |
| Accident & Emergency | 164.16 (133.51) | 31.27 (110.66) | 20.85 (72.77) | 166.73 (134.72) | 36.21 (100.28) | 16.81 (58.61) |
| Ophthalmology Inpatient | 389.06  (1024.06) | 28.84  (165.00) | 31.71  (154.31) | 511.84  (846.10) | 97.22  (332.29) | 25.77  (105.41) |
| Other Inpatient ward | 37.07  (334.16) | 0.00 (0.00) | 8.64  (98.17) | 32.06  (327.09) | 0.01 (0.09) | 0.02  (0.18) |
| Ophthalmology outpatient | 151.37 (361.28) | 180.35 (564.90) | 65.12 (111.95) | 164.93 (344.77) | 173.39 (280.86) | 116.67 (341.87) |
| Other outpatient | 43.58  (149.46) | 44.49 (429.39) | 27.98 (227.85) | 31.81 (74.62) | 15.18 (97.82) | 19.44  (145.52) |
| Other HBS* | 106.17 (21.96) | 3.56 (31.87) | 0.00 (0.00) | 80.96 (16.82) | 4.06 (34.98) | 32.05 (327.26) |

* HBS=hospital-based service
