# Supplementary material for: Economic evaluation of an adjunctive intraocular and peri-ocular steroid vitreoretinal surgery for open globe trauma: Cost-effectiveness of the ASCOT randomised controlled trial
Source: PLoS One. 2024 Dec 16;19(12):e0311158. doi: 10.1371/journal.pone.0311158 (PMC11649106; doi:10.1371/journal.pone.0311158)
Supplement: S2 Table — (DOCX) [file pone.0311158.s002.docx]

# Supporting information

**S2 Table. Summary of community-based service use mean (SD) costs by patients (n=259) in the ASCOT trial over 3 time-points (baseline, 3 months and 6 months).**

|  | **Standard Care (n = 129)** | | | **ASCOT Intervention (n = 130)** | | |
| --- | --- | --- | --- | --- | --- | --- |
|  | Baseline | 3 months | 6 months | Baseline | 3 months | 6 months |
| General practitioner (GP) Visit | 42.83 (77.29) | 49.38  (102.06) | 38.30 (76.05) | 51.46 (95.31) | 49.38 (113.26) | 53.39 (146.80) |
| Practice Nurse | 22.64 (91.17) | 15.12 (53.35) | 20.35 (87.32) | 17.31 (54.08) | 15.00 (61.52) | 15.00 (145.09) |
| District Nurse | 0.66 (7.48) | 5.19 (46.96) | 1.24 (14.89) | 37.69 (400.40) | 17.23 (196.46) | 26.73 (233.29) |
| Social Worker | 0.00 (0.00) | 5.11 (50.74) | 0.00 (0.00) | 20.39 (216.80) | 2.08 (23.68) | 1.39 (11.12) |
| Counsellor | 10.58 (96.31) | 17.91 (126.66) | 27.52 (145.82) | 4.00 (37.80) | 15.31 (107.07) | 12.85 (62.48) |
| Dietician | 0.89 (10.13) | 6.24 (54.38) | 0.00 (0.00) | 0.89 (10.09) | 5.31 (60.52) | 0.00  (0.00) |
| Optician | 13.53 (41.94) | 15.50 (44.22) | 31.98 (97.93) | 20.15 (59.82) | 15.38 (53.95) | 80.73 (724.01) |
| Dentist | 24.03 (59.47) | 17.79 (63.99) | 29.30 (80.86) | 34.19 (67.78) | 10.38 (46.39) | 16.62 (47.59) |
| Physiotherapist | 9.88 (83.02) | 8.99 (78.49) | 7.87 (55.30) | 16.34 (126.75) | 4.46 (30.95) | 15.62 (85.16) |
| Occupational Health Therapist | 6.01 (35.74) | 6.01 (30.04) | 3.61 (23.45) | 12.85 (101.21) | 14.31 (138.43) | 5.96  (44.87) |
| Alternative Therapist | 0.00 (0.00) | 0.00 (0.00) | 0.00 (0.00) | 0.00 (0.00) | 0.00 (0.00) | 0.00  (0.00) |
| Other CBS* | 15.12 (148.40) | 5.58 (52.28) | 10.43 (72.59) | 5.98 (31.69) | 7.65 (45.53) | 17.11 (120.13) |

*CBS= community-based service
