# Supplementary material for: Economic evaluation of an adjunctive intraocular and peri-ocular steroid vitreoretinal surgery for open globe trauma: Cost-effectiveness of the ASCOT randomised controlled trial
Source: PLoS One. 2024 Dec 16;19(12):e0311158. doi: 10.1371/journal.pone.0311158 (PMC11649106; doi:10.1371/journal.pone.0311158)
Supplement: S3 Table — (DOCX) [file pone.0311158.s003.docx]

# Supporting information

**S3 Table.** **Types and cost of medication and drugs administered to patients during the trial.**

| **Best matched drug and name given in PCA** | **Cost per quantity (£)** | **Source** |
| --- | --- | --- |
| Acetazolamide 250mg tablets | 0.12 | PCA 2019 (NHSBSA,2019) |
| Acetazolamide 250mg modified-release capsules | 0.56 | PCA 2019 (NHSBSA,2019) |
| Acular 0.5% eye drops | 0.60 | PCA 2019 (NHSBSA,2019) |
| Revaxis vacc inj 0.5ml pre-filled syringes | 7.80 | PCA 2019 (NHSBSA,2019) |
| Alphagan 0.2% eye drops | 1.37 | PCA 2019 (NHSBSA,2019) |
| Amikacin 500mg/2ml solution for injection vials | 12.00 | PCA 2019 (NHSBSA,2019) |
| Amitriptyline 10mg tablets | 0.03 | PCA 2019 (NHSBSA,2019) |
| Perindopril erbumine 4mg / Amlodipine 5mg tablets | 0.17 | PCA 2019 (NHSBSA,2019) |
| Amoxicillin 250mg capsules | 0.05 | PCA 2019 (NHSBSA,2019) |
| Amoxicillin 500mg capsules | 0.06 | PCA 2019 (NHSBSA,2019) |
| Iopidine 5mg/ml eye drops | 2.18 | PCA 2019 (NHSBSA,2019) |
| Iopidine 1% eye drops 0.25ml unit dose | 3.24 | PCA 2019 (NHSBSA,2019) |
| Cefuroxime 750mg powder for injection vials | 2.52 | PCA 2019 (NHSBSA,2019) |
| Cefuroxime 1.5g powder for injection vials | 5.05 | PCA 2019 (NHSBSA,2019) |
| Cefuroxime 250mg tablets | 1.26 | PCA 2019 (NHSBSA,2019) |
| Atropine 1% eye drops | 13.19 | PCA 2019 (NHSBSA,2019) |
| Atropine 0.5% eye drops | 0.76 | PCA 2019 (NHSBSA,2019) |
| Augmentin 625mg tablets | 0.46 | PCA 2019 (NHSBSA,2019) |
| Azarga 10mg/ml / 5mg/ml eye drops | 2.21 | PCA 2019 (NHSBSA,2019) |
| Azopt 10mg/ml eye drops | 1.38 | PCA 2019 (NHSBSA,2019) |
| Betamethasone valerate 0.1% ointment | 0.06 | PCA 2019 (NHSBSA,2019) |
| Betnesol 0.1% eye/ear/nose drops | 0.23 | PCA 2019 (NHSBSA,2019) |
| Betnesol 500microgram soluble tablets | 1.17 | PCA 2019 (NHSBSA,2019) |
| Bimatoprost 100micrograms/ml eye drops | 3.90 | PCA 2019 (NHSBSA,2019) |
| Bimatoprost 300micrograms/ml eye drops | 3.43 | PCA 2019 (NHSBSA,2019) |
| Brinzolamide 10mg/ml eye drops | 0.44 | PCA 2019 (NHSBSA,2019) |
| Yellox 900micrograms/ml eye drops | 1.70 | PCA 2019 (NHSBSA,2019) |
| Brufen 200mg tablets | 0.04 | PCA 2019 (NHSBSA,2019) |
| Brufen 400mg tablets | 0.08 | PCA 2019 (NHSBSA,2019) |
| Brufen 600mg tablets | 0.12 | PCA 2019 (NHSBSA,2019) |
| Viscotears 2mg/g liquid gel | 0.16 | PCA 2019 (NHSBSA,2019) |
| Viscotears 2mg/g eye gel 0.6ml unit dose | 0.18 | PCA 2019 (NHSBSA,2019) |
| GelTears 0.2% gel | 0.28 | PCA 2019 (NHSBSA,2019) |
| Cefalexin 250mg capsules | 0.06 | PCA 2019 (NHSBSA,2019) |
| Ceftazidime 1g powder for solution for injection vials | 4.50 | PCA 2019 (NHSBSA,2019) |
| Ceftazidime 2g powder for solution for injection vials | 6.58 | PCA 2019 (NHSBSA,2019) |
| Cefuroxime 5% eye drops preservative free | 31.61 | PCA 2019 (NHSBSA,2019) |
| Cefuroxime 5% eye drops | 30.22 | PCA 2019 (NHSBSA,2019) |
| Celluvisc 0.5% eye drops 0.4ml unit dose | 0.16 | PCA 2019 (NHSBSA,2019) |
| Celluvisc 1% eye drops 0.4ml unit dose | 0.12 | PCA 2019 (NHSBSA,2019) |
| Chloramphenicol 1% eye ointment | 0.48 | PCA 2019 (NHSBSA,2019) |
| Chloramphenicol 0.5% eye drops | 0.28 | PCA 2019 (NHSBSA,2019) |
| Ciprofloxacin 250mg tablets | 0.08 | PCA 2019 (NHSBSA,2019) |
| Ciprofloxacin 500mg tablets | 0.10 | PCA 2019 (NHSBSA,2019) |
| Ciprofloxacin 100mg tablets | 0.35 | PCA 2019 (NHSBSA,2019) |
| Ciprofloxacin 750mg tablets | 0.80 | PCA 2019 (NHSBSA,2019) |
| Ciprofloxacin 0.2% eye drops preservative free | 8.71 | PCA 2019 (NHSBSA,2019) |
| Ciprofloxacin 3mg/g eye ointment | 2.75 | PCA 2019 (NHSBSA,2019) |
| Ciloxan 0.3% eye drops | 0.94 | PCA 2019 (NHSBSA,2019) |
| Clindamycin 300mg capsules | 1.27 | PCA 2019 (NHSBSA,2019) |
| Co-amoxiclav 250mg/125mg tablets | 0.09 | PCA 2019 (NHSBSA,2019) |
| Co-amoxiclav 500mg/100mg inj vials | 1.47 | PCA 2019 (NHSBSA,2019) |
| Co-amoxiclav 1000mg/200mg inj vials | 2.21 | PCA 2019 (NHSBSA,2019) |
| Co-amoxiclav 250mg/62mg/5ml oral suspension sugar free | 0.02 | PCA 2019 (NHSBSA,2019) |
| Co-amoxiclav 125mg/31mg/5ml oral suspension | 0.05 | PCA 2019 (NHSBSA,2019) |
| Co-amoxiclav 500mg/125mg tablets | 0.11 | PCA 2019 (NHSBSA,2019) |
| Co-amoxiclav 400mg/57mg/5ml oral suspension sugar free | 0.09 | PCA 2019 (NHSBSA,2019) |
| Co-amoxiclav 875mg/125mg tablets | 1.29 | PCA 2019 (NHSBSA,2019) |
| Co-codamol 8mg/500mg tablets | 0.03 | PCA 2019 (NHSBSA,2019) |
| Codeine 15mg tablets | 0.03 | PCA 2019 (NHSBSA,2019) |
| Codeine 60mg tablets | 0.06 | PCA 2019 (NHSBSA,2019) |
| Codeine 30mg tablets | 0.04 | PCA 2019 (NHSBSA,2019) |
| Co-dydramol 10mg/500mg tablets | 0.03 | PCA 2019 (NHSBSA,2019) |
| Combigan eye drops | 1.98 | PCA 2019 (NHSBSA,2019) |
| Cosopt 20mg/ml / 5mg/ml eye dps 0.2ml ud preservative free | 0.48 | PCA 2019 (NHSBSA,2019) |
| Cyclizine 50mg tablets | 0.07 | PCA 2019 (NHSBSA,2019) |
| Minims cyclopentolate hydrochloride 1% eye dps 0.5ml ud | 0.58 | PCA 2019 (NHSBSA,2019) |
| Minims cyclopentolate hydrochloride 0.5% eye dps 0.5ml ud | 0.57 | PCA 2019 (NHSBSA,2019) |
| Dexafree 1mg/1ml eye drops 0.4ml unit dose | 0.32 | PCA 2019 (NHSBSA,2019) |
| Dexamethasone 0.1% eye dps 0.4ml unit dose preservative free | 0.52 | PCA 2019 (NHSBSA,2019) |
| Maxidex 0.1% eye drops | 0.28 | PCA 2019 (NHSBSA,2019) |
| Diamox SR 250mg capsules | 0.56 | PCA 2019 (NHSBSA,2019) |
| Dorzolamide 20mg/Timolol 5mg/ml eye dps 0.2ml preserv free | 0.48 | PCA 2019 (NHSBSA,2019) |
| Doxycycline 100mg capsules | 0.11 | PCA 2019 (NHSBSA,2019) |
| Dropodex 0.1% eye drops 0.4ml unit dose | 0.52 | PCA 2019 (NHSBSA,2019) |
| DuoTrav 40micrograms/ml / 5mg/ml eye drops | 5.55 | PCA 2019 (NHSBSA,2019) |
| Exocin 0.3% eye drops | 0.43 | PCA 2019 (NHSBSA,2019) |
| Fentanyl 100micrograms/hour transdermal patches | 11.57 | PCA 2019 (NHSBSA,2019) |
| Flucloxacillin 500mg capsules | 0.09 | PCA 2019 (NHSBSA,2019) |
| FML Liquifilm 0.1% ophthalmic suspension | 0.33 | PCA 2019 (NHSBSA,2019) |
| Prednisolone sodium phosphate 0.5% ear/eye drops | 0.23 | PCA 2019 (NHSBSA,2019) |
| PF Drops Sodium Hyaluronate 0.15% eye drops preservative free | 0.70 | PCA 2019 (NHSBSA,2019) |
| Levofloxacin 5mg/ml eye drops | 1.43 | PCA 2019 (NHSBSA,2019) |
| Pred Forte 1% eye drops | 0.36 | PCA 2019 (NHSBSA,2019) |
| Timolol 0.25% eye drops | 0.21 | PCA 2019 (NHSBSA,2019) |
| Tobradex 3mg/ml / 1mg/ml eye drops | 1.07 | PCA 2019 (NHSBSA,2019) |
| Travatan 40micrograms/ml eye drops | 4.38 | PCA 2019 (NHSBSA,2019) |
| Gabapentin 300mg capsules | 0.03 | PCA 2019 (NHSBSA,2019) |
| Ganfort 0.3mg/ml / 5mg/ml eye drops | 4.70 | PCA 2019 (NHSBSA,2019) |
| Gentamicin 0.3% ear/eye drops | 0.27 | PCA 2019 (NHSBSA,2019) |
| Glycopyrronium bromide 200micrograms/5ml oral suspension | 0.11 | PCA 2019 (NHSBSA,2019) |
| Moxivig 0.5% eye drops | 1.96 | PCA 2019 (NHSBSA,2019) |
| Hylo-Tear 0.1% eye drops preservative free | 0.85 | PCA 2019 (NHSBSA,2019) |
| Latanoprost 50micrograms/ml eye drops | 2.75 | PCA 2019 (NHSBSA,2019) |
| Lumigan 100micrograms/ml eye drops | 3.90 | PCA 2019 (NHSBSA,2019) |
| Maxitrol eye drops | 0.34 | PCA 2019 (NHSBSA,2019) |
| Monopost 50micrograms/ml eye drops 0.2ml unit dose | 0.28 | PCA 2019 (NHSBSA,2019) |
| Simbrinza 10mg/ml / 2mg/ml eye drops | 1.85 | PCA 2019 (NHSBSA,2019) |
| Sodium chloride 0.9% eye drops | 0.80 | PCA 2019 (NHSBSA,2019) |
| Hylo-Forte 0.2% eye drops preservative free | 0.95 | PCA 2019 (NHSBSA,2019) |
| Hypromellose 0.3% eye drops | 0.13 | PCA 2019 (NHSBSA,2019) |
| Ibuprofen 400mg tablets | 0.04 | PCA 2019 (NHSBSA,2019) |
| Diamox Sodium Parenteral 500mg powder for solution for injection vials (Advanz Pharma) | 14.76 | NICE, 2020 BNF |
| Ketorolac 0.5% eye drops | 0.72 | PCA 2019 (NHSBSA,2019) |
| Lacri-lube eye ointment | 0.81 | PCA 2019 (NHSBSA,2019) |
| Lamotrigine 200mg tablets | 0.25 | PCA 2019 (NHSBSA,2019) |
| Lansoprazole 30mg gastro-resistant capsules | 0.04 | PCA 2019 (NHSBSA,2019) |
| Levofloxacin 500mg tablets | 2.35 | PCA 2019 (NHSBSA,2019) |
| Lotemax 0.5% eye drops | 1.10 | PCA 2019 (NHSBSA,2019) |
| Blephasol lotion | 0.08 | PCA 2019 (NHSBSA,2019) |
| Moxifloxacin 400mg tablets | 2.03 | PCA 2019 (NHSBSA,2019) |
| Maxitrol eye ointment | 0.41 | PCA 2019 (NHSBSA,2019) |
| Mydrilate 0.5% solution | 1.62 | PCA 2019 (NHSBSA,2019) |
| Mydrilate 1% solution | 1.62 | PCA 2019 (NHSBSA,2019) |
| Morphine sulfate 10mg/5ml oral solution | 0.02 | PCA 2019 (NHSBSA,2019) |
| Naproxen 500mg tablets | 0.28 | PCA 2019 (NHSBSA,2019) |
| Natacyn 5% ophthalmic suspension | 56.00 | PCA 2019 (NHSBSA,2019) |
| Nevanac 1mg/ml eye drops | 2.98 | PCA 2019 (NHSBSA,2019) |
| Nevanac 1mg/ml eye drops1mg/ml eye drops | 2.98 | PCA 2020 (NHSBSA,2019) |
| VitA-POS eye ointment preservative free | 0.55 | PCA 2019 (NHSBSA,2019) |
| Oftaquix 5mg/ml eye drops 0.3ml unit dose | 0.60 | PCA 2019 (NHSBSA,2019) |
| Olopatadine 1mg/ml eye drops | 0.94 | PCA 2019 (NHSBSA,2019) |
| Omeprazole 20mg gastro-resistant capsules | 0.03 | PCA 2019 (NHSBSA,2019) |
| Omeprazole 40mg gastro-resistant capsules | 0.10 | PCA 2019 (NHSBSA,2019) |
| Ondansetron 4mg tablets | 0.68 | PCA 2019 (NHSBSA,2019) |
| Ondansetron 8mg tablets | 0.51 | PCA 2019 (NHSBSA,2019) |
| Opatanol 1mg/ml eye drops | 0.94 | PCA 2019 (NHSBSA,2019) |
| Optive 0.5% eye drops | 0.75 | PCA 2019 (NHSBSA,2019) |
| Paracetamol 1g tablets | 0.02 | PCA 2019 (NHSBSA,2019) |
| Paracetamol 500mg tablets | 0.02 | PCA 2019 (NHSBSA,2019) |
| Prednisolone 10mg tablets | 0.06 | PCA 2019 (NHSBSA,2019) |
| Prednisolone 20mg tablets | 0.13 | PCA 2019 (NHSBSA,2019) |
| Oramorph 20mg/ml concentrated oral solution | 0.16 | PCA 2019 (NHSBSA,2019) |
| Oramorph 10mg/5ml oral solution | 0.02 | PCA 2019 (NHSBSA,2019) |
| Minims oxybuprocaine hydrochloride 0.4% eye dps 0.5ml ud | 0.53 | PCA 2019 (NHSBSA,2019) |
| Minims phenylephrine hydrochloride 2.5% eye dps 0.5ml ud | 0.59 | PCA 2019 (NHSBSA,2019) |
| Ramipril 10mg capsules | 0.04 | PCA 2019 (NHSBSA,2019) |
| Saflutan 15micrograms/ml eye drops 0.3ml unit dose | 0.41 | PCA 2019 (NHSBSA,2019) |
| Sertraline 100mg tablets | 0.05 | PCA 2019 (NHSBSA,2019) |
| Sodium chloride 5% eye drops (drug) | 2.52 | PCA 2019 (NHSBSA,2019) |
| Dexamethasone (base) 3.3mg/1ml inj ampoules | 2.31 | PCA 2019 (NHSBSA,2019) |
| Thealoz Duo eye drops preservative free | 0.90 | PCA 2019 (NHSBSA,2019) |
| Tetanus immunoglobulin human 250unit inj vials | 170.00 | PCA 2019 (NHSBSA,2019) |
| Timolol 0.5% eye drops | 0.21 | PCA 2019 (NHSBSA,2019) |
| Timoptol 0.5% eye drops | 0.62 | PCA 2019 (NHSBSA,2019) |
| Tramadol 50mg capsules | 0.03 | PCA 2019 (NHSBSA,2019) |
| Minims tropicamide 1% eye drops 0.5ml unit dose | 0.57 | PCA 2019 (NHSBSA,2019) |
| Trusopt 20mg/ml eye drops 0.2ml unit dose preservative free | 0.40 | PCA 2019 (NHSBSA,2019) |
| Vancomycin 500mg powder for solution for infusion vials | 5.80 | PCA 2019 (NHSBSA,2019) |
| Vancomycin 1g powder for solution for infusion vials | 11.59 | PCA 2019 (NHSBSA,2019) |
| Voriconazole 200mg tablets | 23.60 | PCA 2019 (NHSBSA,2019) |
| Voriconazole 50mg tablets | 6.38 | PCA 2019 (NHSBSA,2019) |
| Voriconazole 1% eye drops preservative free | 96.18 | PCA 2019 (NHSBSA,2019) |
| Voriconazole 1% eye drops | 76.12 | PCA 2019 (NHSBSA,2019) |
| Voriconazole 100mg tablets | 9.85 | PCA 2019 (NHSBSA,2019) |
| Xalatan 50micrograms/ml eye drops | 3.88 | PCA 2019 (NHSBSA,2019) |
| Zomorph 10mg modified-release capsules | 0.06 | PCA 2019 (NHSBSA,2019) |
