# Supplementary material for: Economic evaluation of an adjunctive intraocular and peri-ocular steroid vitreoretinal surgery for open globe trauma: Cost-effectiveness of the ASCOT randomised controlled trial
Source: PLoS One. 2024 Dec 16;19(12):e0311158. doi: 10.1371/journal.pone.0311158 (PMC11649106; doi:10.1371/journal.pone.0311158)
Supplement: S4 Table — (DOCX) [file pone.0311158.s004.docx]

# Supporting information

**S4 Table.** **Distribution for the probabilistic sensitivity analysis.**

| **Parameters** | **Mean** | **Standard error** | **Distribution** |
| --- | --- | --- | --- |
| Standard Care |  |  |  |
| Probability ETDRS ≥ 10 | 0.434 |  | Beta |
| Probability ETDRS < 10 | 0.566 |  | Beta |
| Cost ≥ 10 | £5,268 | £929 | Gamma |
| Cost < 10 | £4,969 | £713 | Gamma |
| QALY ≥10 | 0.031 | 0.010 | Beta |
| QALY < 10 | 0.026 | 0.011 | Beta |
| VFQ ≥10 | 0.348 | 0.012 | Beta |
| VFQ < 10 | 0.342 | 0.014 | Beta |
|  |  |  |  |
| ASCOT Intervention |  |  |  |
| Probability ETDRS ≥ 10 | 0.469 |  | Beta |
| Probability ETDRS < 10 | 0.530 |  | Beta |
| Cost ≥ 10 | £6,447 | £708 | Gamma |
| Cost <10 | £4,713 | £559 | Gamma |
| QALY ≥10 | 0.045 | 0.013 | Beta |
| QALY < 10 | 0.034 | 0.013 | Beta |
| VFQ ≥10 | 0.340 | 0.013 | Beta |
| VFQ < 10 | 0.349 | 0.012 | Beta |
